# Dynamic structure and composition of bone investigated by nanoscale infrared spectroscopy

**DOI:** 10.1371/journal.pone.0202833

**Published:** 2018-09-04

**Authors:** Laurianne Imbert, Samuel Gourion-Arsiquaud, Eduardo Villarreal-Ramirez, Lyudmila Spevak, Hayat Taleb, Marjolein C. H. van der Meulen, Richard Mendelsohn, Adele L. Boskey

**Affiliations:** 1 Hospital for Special Surgery, Research Institute, New York, New York, United States of America; 2 TRI Princeton, Princeton, New Jersey, United States of America; 3 Tissue Bioengineering Laboratory, DEPeI, Faculty of Dentistry, National Autonomous University of Mexico, Mexico Distrito Federal, Mexico; 4 Sibley School of Mechanical and Aerospace Engineering, Cornell University, Ithaca, New York, United States of America; 5 Meinig School of Biomedical Engineering, Cornell University, Ithaca, New York, United States of America; 6 Department of Chemistry, Newark College of Arts and Science, Rutgers University, New Jersey, United States of America; 7 Department of Biochemistry, Weill Cornell Medicine, New York, New York, United States of America; University of South Carolina, UNITED STATES

## Abstract

Bone is a highly organized tissue in which each structural level influences the macroscopic and microscopic mechanical behavior. In particular, the quantity, quality, and distribution of the different bone components, i.e. collagen matrix and hydroxyapatite crystals, are associated with bone strength or fragility. Common spectroscopic techniques used to assess bone composition have resolutions limited to the micrometer range. In this study, our aims were two-fold: i) to develop and validate the AFM-IR methodology for skeletal tissues and ii) to apply the methodology to sheep cancellous bone with the objective to obtain novel findings on the composition and structure of trabecular packets.To develop the methodology, we assessed spatial and temporal reproducibility using a known homogeneous material (polymethylmethacrylate, PMMA). We verified that the major peak positions were similar and not shifted when compared to traditional Fourier Transform Infrared imaging (FTIRI). When AFM-IR was applied to sheep cancellous bone, the mineral-to-matrix ratio increased and the acid phosphate substitution ratio decreased as a function of tissue maturity. The resolution of the technique enabled visualization of different stages of the bone maturation process, particularly newly-formed osteoid prior to mineralization. We also observed alternating patterns of IR parameters in line and imaging measurements, suggesting the apposition of layers of alternating structure and / or composition that were not visible with traditional spectroscopic methods. In conclusion, nanoscale IR spectroscopy demonstrates novel compositional and structural changes within trabecular packets in cancellous bone. Based on these results, AFM-IR is a valuable tool to investigate cancellous bone at the nanoscale and, more generally, to analyze small dynamic areas that are invisible to traditional spectroscopic methods.

## Introduction

Bone is a highly structured and dynamic heterogeneous living material with a complex multiscale organization, as revealed by extensive investigations [1,2]. At the macroscopic scale, one distinguishes two types of bone tissue, cortical and cancellous, each composed of repeating microstructural units, termed osteons and trabecular packets, respectively. These units are composed of lamella consisting of mineralized collagen fibers that are composed of collagen fibrils at the nanostructural level, in turn formed from collagen molecules with hydroxyapatite crystals deposited on them. Ultrastructural analyses have described the arrangement and orientation of the mineralized collagen fibril [2–4]. The nanostructure and ultrastructure determine the mechanical properties at higher scales [5–8]. In particular, the quality and distribution of the bone constituents are associated with bone strength or fragility [9], as evident in skeletal diseases, including Osteogenesis Imperfecta [10–13] and osteoporosis [14,15]. Moreover, the individual properties of the mineral and collagen, and their interactions, play a role in determining the mechanical properties within osteons [16] and trabecular packets [17].

The techniques commonly used to study bone composition include Fourier Transform Infrared spectroscopy (FTIR) [9,18–22], Raman spectroscopy [23,24], and the corresponding imaging techniques, Fourier transform Infrared imaging (FTIRI) and Raman imaging. Both FTIR and Raman spectroscopy are based on the vibrational states of the molecular species that comprise the material, but they detect different interactions of light with the sample, absorption and scattering, respectively. Bone quality parameters can be derived from the resulting spectra [18]. Both techniques produce maps or point-by-point measurements with spatial resolutions limited to the micrometer range. Therefore, novel techniques are needed to investigate extremely heterogeneous samples at the nanoscale.

Recently, several techniques have been developed to study material composition at the nanoscale, including scattering scanning near-field optical microscopy (s-SNOM), tip-enhanced Raman spectroscopy (TERS), and AFM-IR. S-SNOM and TERS measure the light scattered by the sample, whereas AFM-IR measures the absorbed light, which confers several advantages compared with the scattering techniques. In particular, AFM-IR does not require special probes, unlike TERS, or specific optical properties, unlike s-SNOM [25–28].

**Fig 1:**
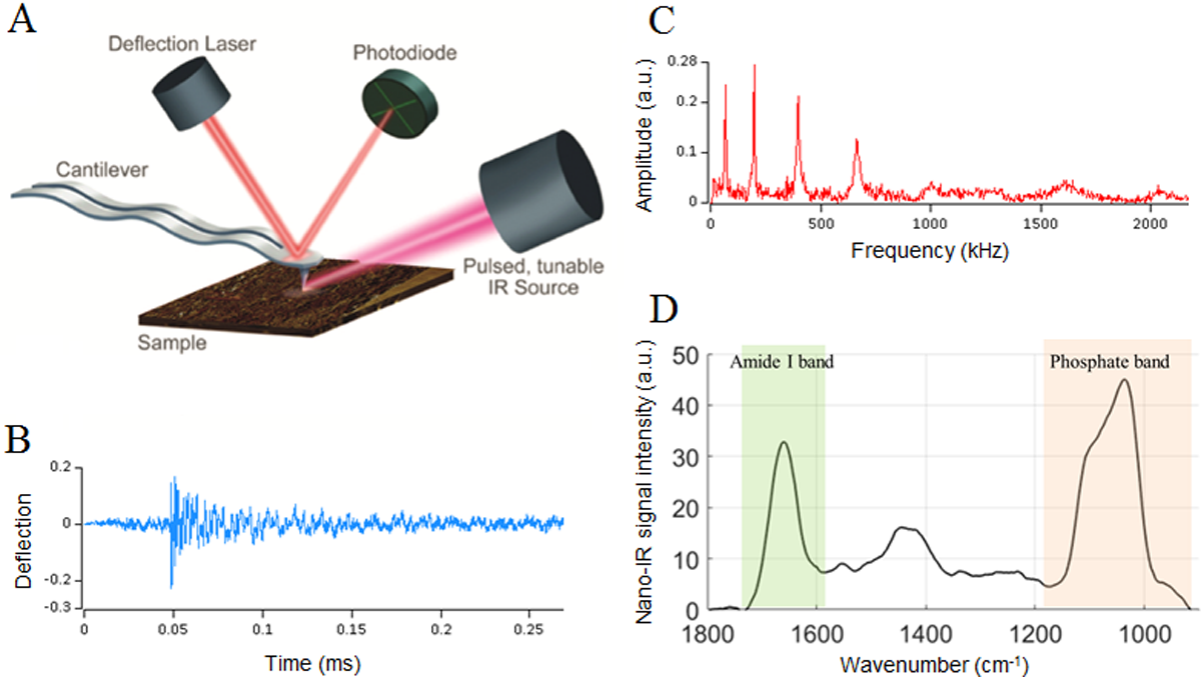
Principle of AFM-IR. (A) The sample surface is illuminated with a tunable IR laser. When the wavelength of the laser corresponds to an IR absorption band of the sample, the IR absorption will create heat and entail a rapid thermal expansion of the absorbing regions. This rapid thermal expansion induces oscillations (B) in the AFM tip at its resonant frequencies (C). Because the absorption is proportional to the cantilever oscillation, an absorption spectrum can be derived. (D) Absorption spectrum acquired on cancellous bone in which the phosphate band (920–1200 cm^-1^) and the amide I band (1592–1712 cm^-1^) characterize the mineral and collagen components, respectively. Adapted with permission from Anasys Instruments.

AFM-IR is a novel technique developed by Dazzi *et al*. [26,29,30] that combines atomic force microscopy (AFM) and infrared (IR) techniques to overcome the diffraction limit and enable acquisition of IR spectra with spatial resolution in the nanometer range (Fig 1). In brief, the sample surface is illuminated with a tunable IR laser. If the wavelength of the laser corresponds to the IR absorption band of the sample, the IR absorption will create heat, leading to rapid thermal expansion of the absorbing regions. This rapid thermal expansion induces oscillations in the AFM tip at its contact-resonance frequencies. The absorption is proportional to the cantilever oscillation, enabling a nanomechanical IR absorption spectrum to be derived. This technique has been used mainly in the polymer field [25,31–34], but applications related to biological materials are growing including the mapping of viruses, bacteria, and cells [25,35–38], analysis of protein conformation [28,39–40], and studies of tissue composition. For instance, the distribution of lipids was mapped in the stratum corneum [41]. Our group previously demonstrated the feasibility of the technique for bone compositional analyses using a single baboon femur [42].

In the present study, we aimed to create a framework for the use of AFM-IR on bone tissue and investigate the nanoscale properties of sheep trabecular bone. The reproducibility of the technique was initially assessed on polymethylmethacrylate (PMMA), a homogeneous resin. We validated the methodology by comparing data acquired with AFM-IR and regular FTIR on PMMA and bone samples. Using AFM-IR on cancellous bone showed a reproducible alternating pattern suggesting apposition of layers of different composition and/or structure that was not visible with the microscale resolution of traditional spectroscopic techniques.

## Materials and methods

### Methodology development and validation

#### AFM-IR data collection and processing

Data were acquired in contact mode using the third resonant mode of a gold-coated AFM cantilever (tip radius <25 nm, resonant frequency 13±4 kHz, spring constant 0.07–0.4 N/m, cantilever model PR-EX-nIR2-10, Anasys Instruments, Santa Barbara, CA, USA) on an AFM-IR instrument (nano-IR2, Anasys Instruments, Santa Barbara, CA, USA). A frequency window of 60 kHz was used. The tunable mid-IR OPO laser (EKSPLA, Lithuania) produced laser pulses of 10 ns at a frequency of 1 kHz. The IR power level incident on the sample was about 0.5 mW, and the focused laser spot size was about 100 μm. With this configuration (IR laser, cantilever, and tip), the spatial resolution can reach 50–100 nm.

AFM-IR spectra were collected over a range of 900–1800 cm^-1^, with a spectral resolution of 4 cm^-1^ and an accumulation of 256 scans for each point. Five repeats were acquired for each point. IR absorption images were collected at 6 different wavenumbers (1690, 1660, 1128, 1096, 1030 and 1020 cm^-1^) at a scan rate of 0.1 Hz, with an accumulation of 16 scans at each position. Data were collected using Analysis Studio software (Anasys Instruments, Santa Barbara, CA, USA).

AFM-IR data were analyzed using custom software (version R2016a, MATLAB, Mathworks, Natick, MA, USA). First, all spectra were smoothed using a Savitzky-Golay filter (polynomial order 3). Then each spectrum was baselined using a linear interpolation. For each data point, the average spectrum was calculated by computing the mean IR peak value for each wavenumber. An averaged PMMA spectrum was used to subtract the PMMA contribution from the bone spectra. Commonly used parameters (Table 1) were derived from the spectra and images. The mineral-to-matrix ratio image from AFM-IR was derived as a ratio of intensities at specific wavenumbers (1030 cm^-1^ and 1660 cm^-1^) rather than the ratio of areas used in FTIR.

**Table 1:** Infrared parameters commonly used to analyze bone tissue.

Parameter	Region analyzed (cm^-1^ / cm^-1^)	Meaning
Mineral/matrix ratio (phosphate ν1ν3 / Amide I)	920–1200 / 1596–1712 +	Mineral content relatively to matrix (collagen) content
Crystallinity	1030 / 1020 *	Size and perfection of the hydroxyapatite crystals
Acid phosphate substitution ratio	1128 / 1096 *	Quantity of acid phosphate substituted into the crystal lattice
Collagen maturity	1660 / 1690 *	Maturity of the collagen enzymatic crosslinks

+ Ratio of areas under the bands,

* Ratio of intensities at the indicated wavenumbers

#### Reproducibility assessment on PMMA

Temporal reproducibility was assessed by comparing repeated acquisitions at the same location, and spatial reproducibility was assessed by comparing the mean spectra of adjacent data points. A 4x4 grid of points with a spacing of 5 μm was acquired on 7 sections of PMMA. To assess the temporal reproducibility we calculated and plotted the standard deviation of the 5 repetitions. To assess the spatial reproducibility we calculated and plotted the standard deviation for the averaged spectra of the 16 data points.

#### Comparison to FTIRI

To compare AFM-IR with FTIRI, a known standard method, data from 5 PMMA and bone samples were compared between the two techniques. First AFM-IR spectra were recorded. Then, an area of at least 200 x 200 μm, including the area investigated by AFM-IR (15x15 microns on PMMA and about 40x40 (± 15) microns on bone samples), was recorded by FTIRI (Spotlight 300 Imaging System, Perkin Elmer, Norwalk, CT, USA). Images were collected in transmission mode from 800 cm^-1^ to 2000 cm^-1^ at 4 cm^-1^ spectral resolution and 6.25 μm spatial resolution. The FTIRI images and spectra were processed using ISYS Chemical Imaging analysis software (Spectral Dimensions (presently Malvern), Olney, MD, USA), as detailed elsewhere [43]. The spectra and images obtained by AFM-IR and FTIRI were superimposed and qualitatively compared, focusing on the peak assignments commonly used for FTIRI (Table 1).

### Acquisition on cancellous bone samples

#### Sample preparation

Vertebral biopsies from ovariectomized (OVX) sheep from a prior study were used to validate the proposed methodology. The animals were mature Swiss Rambouillet ewes (6–7 years old, weighing 69–82 kg). Previously, the samples had been fixed, dehydrated, and embedded in PMMA. Transverse thin sections (300 nm) were cut using an ultramicrotome (Reichert-Jung Ultracut E, Vienna, Austria) equipped with a diamond knife (Diatome Ltd., Bienne, Switzerland). These sections, composed of PMMA and cancellous bone, were then transferred onto ZnS flats and mounted onto the sample stage of the AFM-IR instrument. For FTIR analysis, thicker transverse sections (1 μm) were cut using the same ultramicrotome (Reichert-Jung Ultracut E, Vienna, Austria). The sections were transferred to BaF_2_ windows and mounted on the stage of the FTIRI instrument.

#### AFM-IR acquisition

For the AFM-IR analysis, nine regions of interest per cancellous bone sample (3 areas/section, 3 sections/sample), including the trabecular edge and more mature interior bone tissue, were identified, and AFM topography images were acquired. Nine line scans were acquired with 1 μm spacing between points and areal images of one region were acquired per cancellous bone sample.

### Imaging bone collagen after demineralization

To investigate the effect of matrix composition, a single section was demineralized using ethylene diamine tetra-acetic acid (EDTA, 0.5M). To maintain the integrity of the thin section, a drop of EDTA was placed on the section for 24 h and washed off with deionized water. This process was repeated twice over 3 days. Demineralization was confirmed by spectroscopy using the phosphate peak at 1030 cm^-1^, then collagen at 1660 cm^-1^ was examined.

## Results

### Methodology development and validation

#### Reproducibility over time

**Fig 2:**
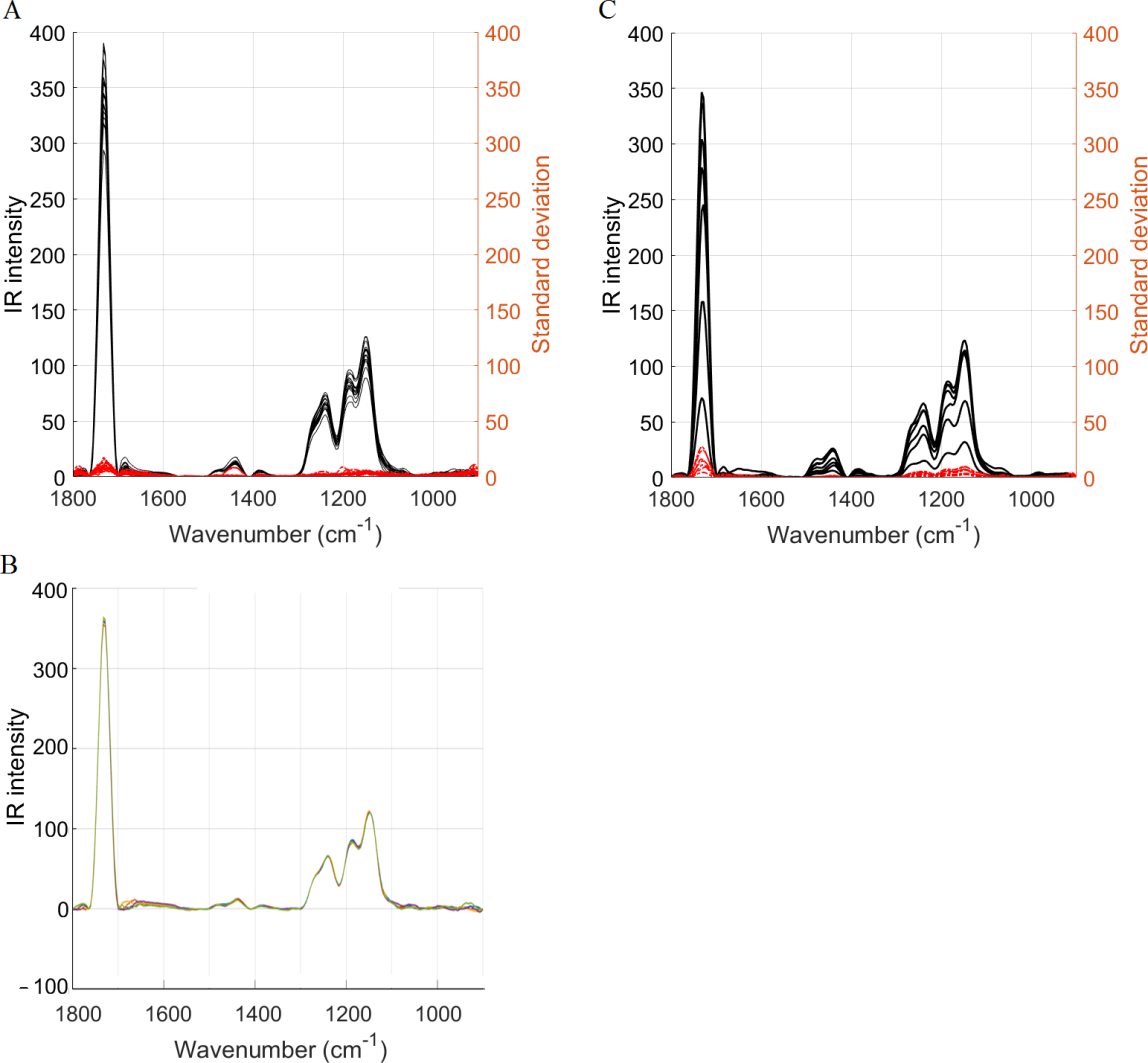
Temporal and spatial reproducibility. (A) The temporal reproducibility was assessed on PMMA (n = 7). The 5 repeated measurements for each of the 16 locations were averaged (black) and the corresponding standard deviation was derived for each location (red). The low standard deviation validates a good temporal reproducibility. (B) Example of a group of five repeated measurements showing a good temporal reproducibility. (C) The spatial reproducibility was assessed on seven samples. The 16 spectra (locations) per sample were averaged (black) and the corresponding standard deviation was derived (red). The low standard deviation validates a good spatial reproducibility.

For all seven PMMA samples, the repeated acquisitions at each point were very similar. The low standard deviation for all wavenumbers (Fig 2A and 2B) validated the temporal reproducibility for a homogeneous sample.

#### Reproducibility in space

For all seven PMMA samples, the standard deviation computed at each wavenumber was low among the 16 points examined (Fig 2C), validating the spatial reproducibility for the homogeneous PMMA.

#### AFM-IR vs FTIR

**Fig 3:**
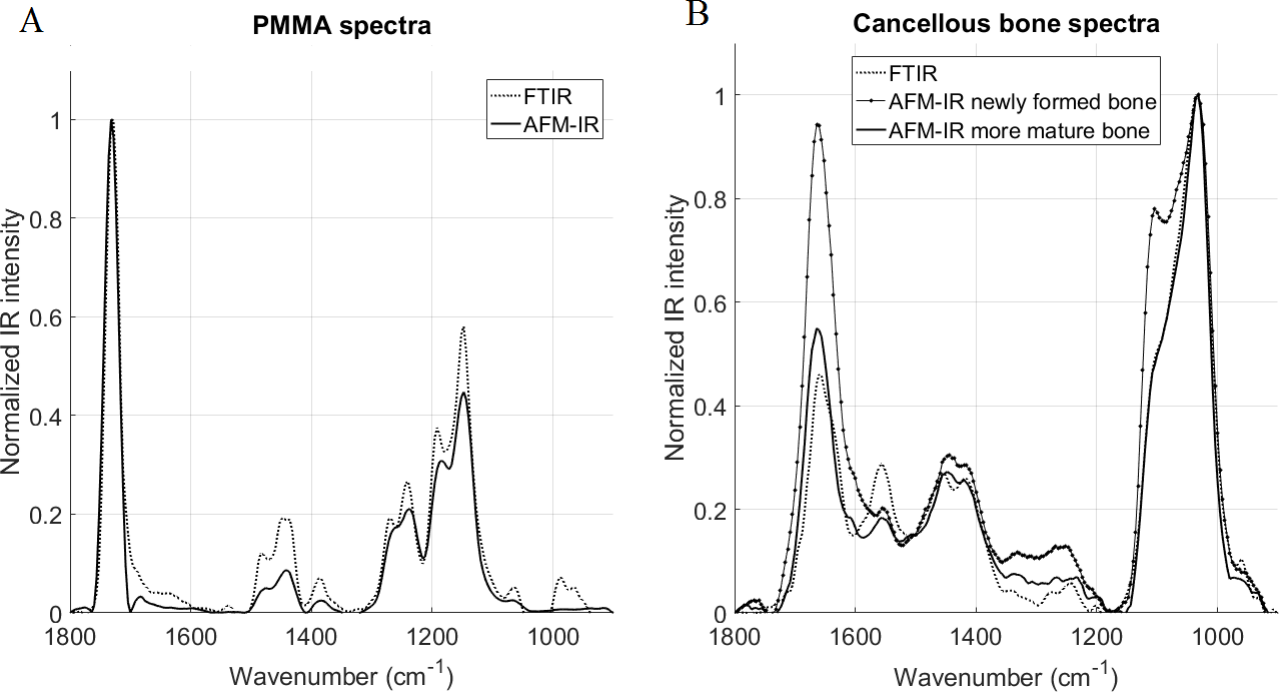
Comparison of normalized FTIR and AFM-IR spectra. Spectra acquired on (A) PMMA and (B) cancellous bone. In the cancellous bone, the newly formed bone was located on the trabecular edge (in the first 20 microns) and more mature bone was closer to the center of the trabecula. No major shifts were observed between the two techniques, but differences in shapes and ratios are evident. In the amide band of the bone spectra (B), the amide II peak was significantly smaller in the AFM-IR spectra and a shoulder was evident around 1600 cm^-1^ not present in the FTIR spectra. In the mineral band for the newly formed bone, the shoulder corresponding to the peak 1128 cm^-1^ was more prominent.

For five samples, FTIR spectra and images were compared to AFM-IR spectra and images. For the homogenous PMMA resin, spectra obtained by the two methods were very similar (Fig 3A). For the bone samples (Fig 3B), the spectra also appeared to be very similar, particularly in the mature bone, with no significant shift of the major peaks (amide I and phosphate). However, interesting differences could be observed. The amide II peak intensity was significantly lower in the AFM-IR spectra compared to FTIR (Fig 3B). The shoulder seen around 1600 cm^-1^ in the AFM-IR was not present in the FTIR spectra, although the peak shape of the AFM-IR spectra did vary. The shape of the ν_1_PO_4_ peak (mineral band) differed from the shape evident in the FTIR spectra. For instance, for the newly-formed bone, a shoulder corresponding to the peak at 1128 cm^-1^ was more prominent.

**Fig 4:**
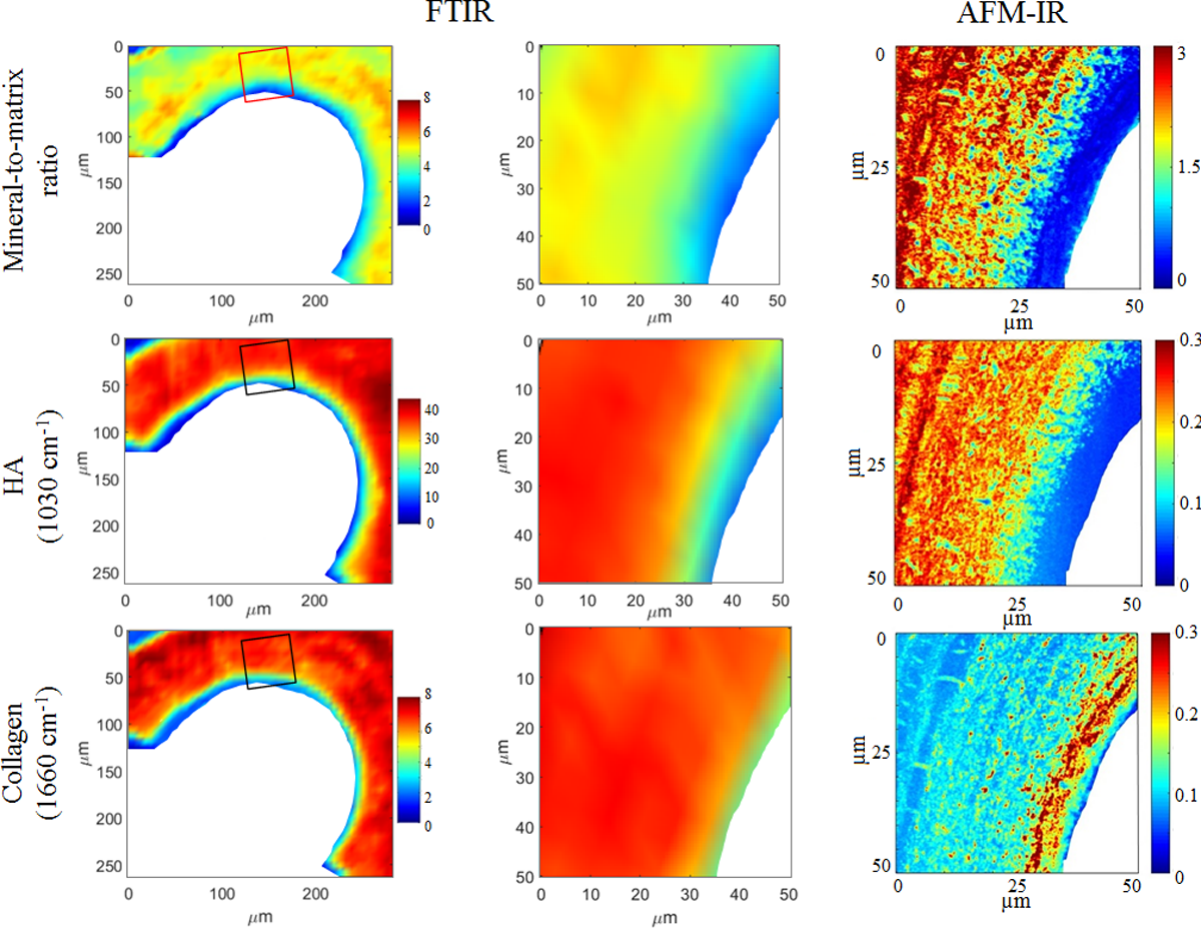
AFM-IR images show a non-mineralized layer (osteoid) not observed with FTIR. Images acquired on the same cancellous bone samples with AFM-IR (right column) and FTIR (left and middle columns). The images in the left column were acquired with FTIR; the rectangles indicate the areas where the corresponding AFM-IR images were acquired. The FTIR images obtained within the rectangular area are enlarged in the middle column to match the size of the AFM-IR images, shown in the right column. The first row is the mineral-to-matrix ratio, the second row shows maps of the hydroxyapatite crystals acquired at 1030 cm^-1^, and the third row shows maps of the collagen acquired at 1660 cm^-1^. Color scales indicate the relative IR intensity for the technique used (left and middle columns share the same color scales).

Due to the large difference in spatial resolution (50–100 nm for AFM-IR vs 6.25 μm for FTIRI), the AFM-IR images were more detailed (Fig 4). In particular, the gradient of collagen content (Fig 4 bottom row) decreased from the trabecular edge in the AFM-IR, while the decreasing gradient was absent in the FTIRI images. This last observation highlights the potential of AFM-IR to investigate small-scale dynamic phenomena such as bone formation.

### Cancellous bone

#### IR parameters vary with bone maturity

**Fig 5:**
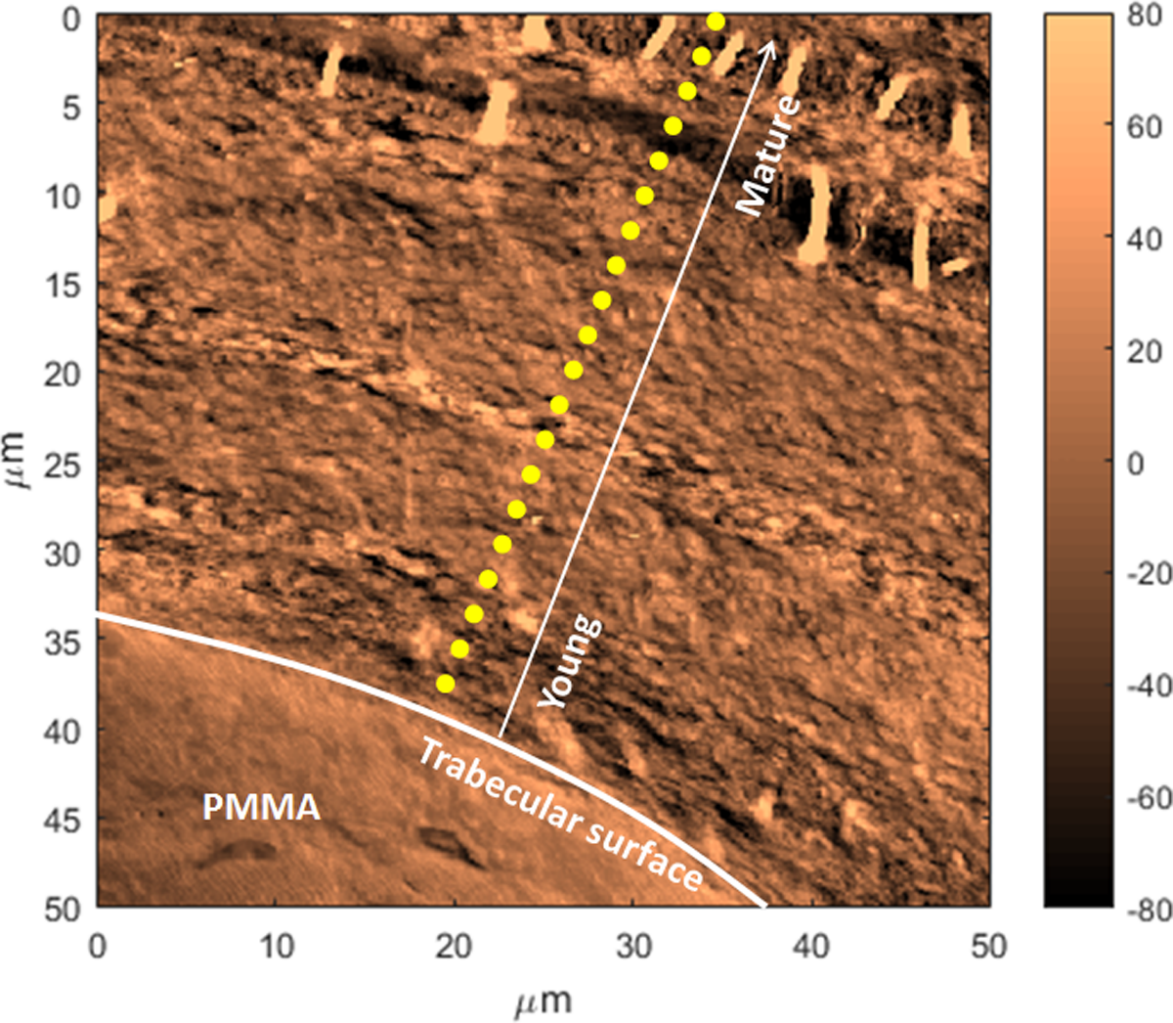
AFM image showing a line scan acquisition. Example of line scan measurements (yellow dotted line) performed from the trabecular surface where the bone tissue is the youngest (the white arrow represents increasing tissue age from the surface), to the interior where the bone tissue is more mature. An ultrastructure is evident with layers of different orientations. Also fibers are visible at the interior, possibly mineralized fibers that appeared when the section was cut and deposited onto the substrate.

**Fig 6:**
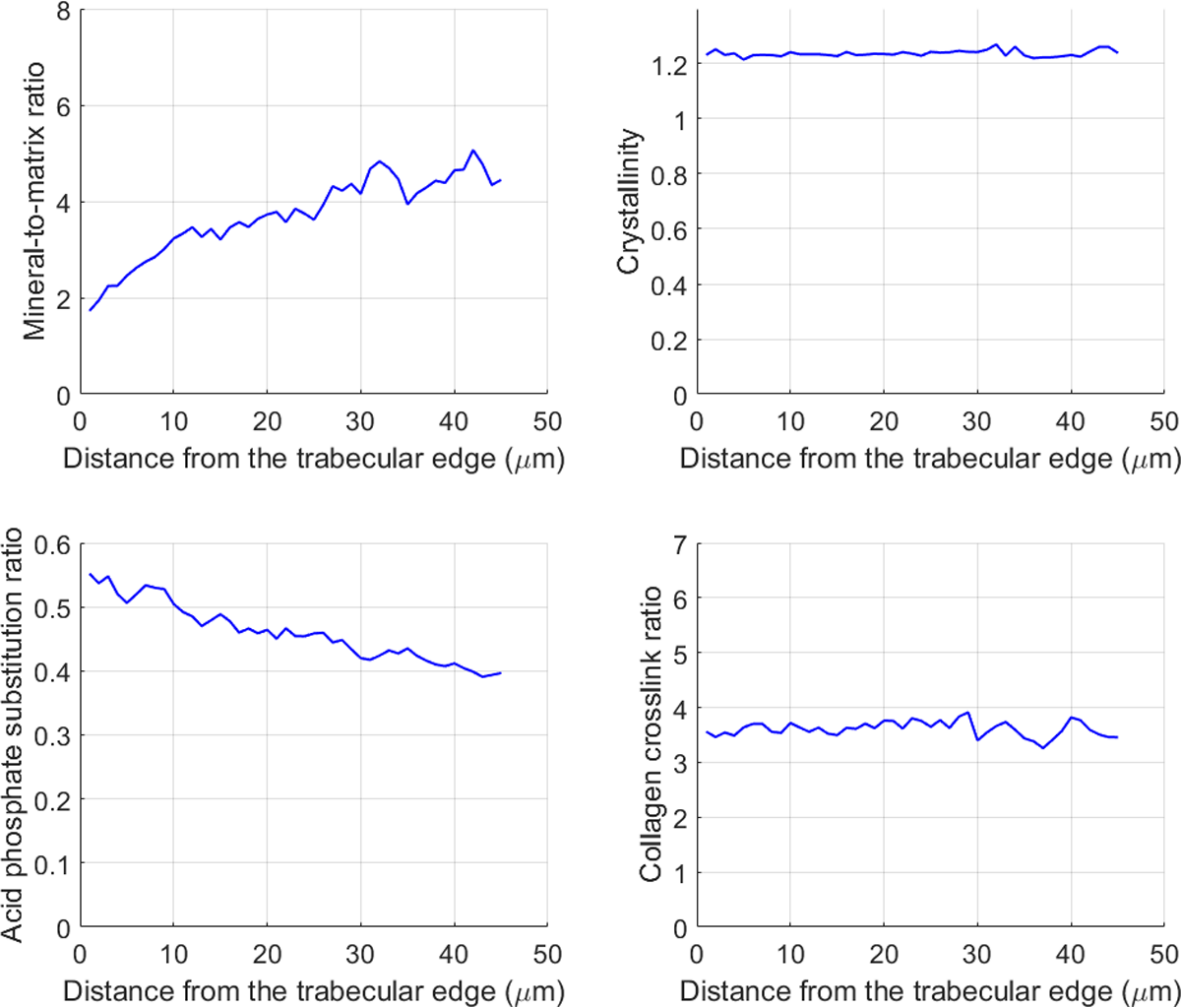
Average (n = 8) of the spectroscopic parameters recorded by AFM-IR as a function of the distance from the trabecular surface. Repeated measurements were acquired every 1 um as a line orthogonal to the trabecular surface. The mineral-to-matrix ratio increased and the acid phosphate substitution ratio decreased with bone maturity. The crystallinity and the collagen crosslink ratio remained constant.

The line scans acquired with AFM-IR for the sheep vertebral samples showed variations with bone maturity, proceeding from the trabecular surface, where the bone is the youngest, to the inside of the trabecula, where bone is more mature (Fig 5). In particular, the mineral-to-matrix ratio increased and the acid phosphate substitution decreased with bone maturity (Fig 6). The AFM-IR images (Fig 4) showed the same variations with bone maturity as those seen in the line scans.

#### Alternating mineralization pattern

**Fig 7:**
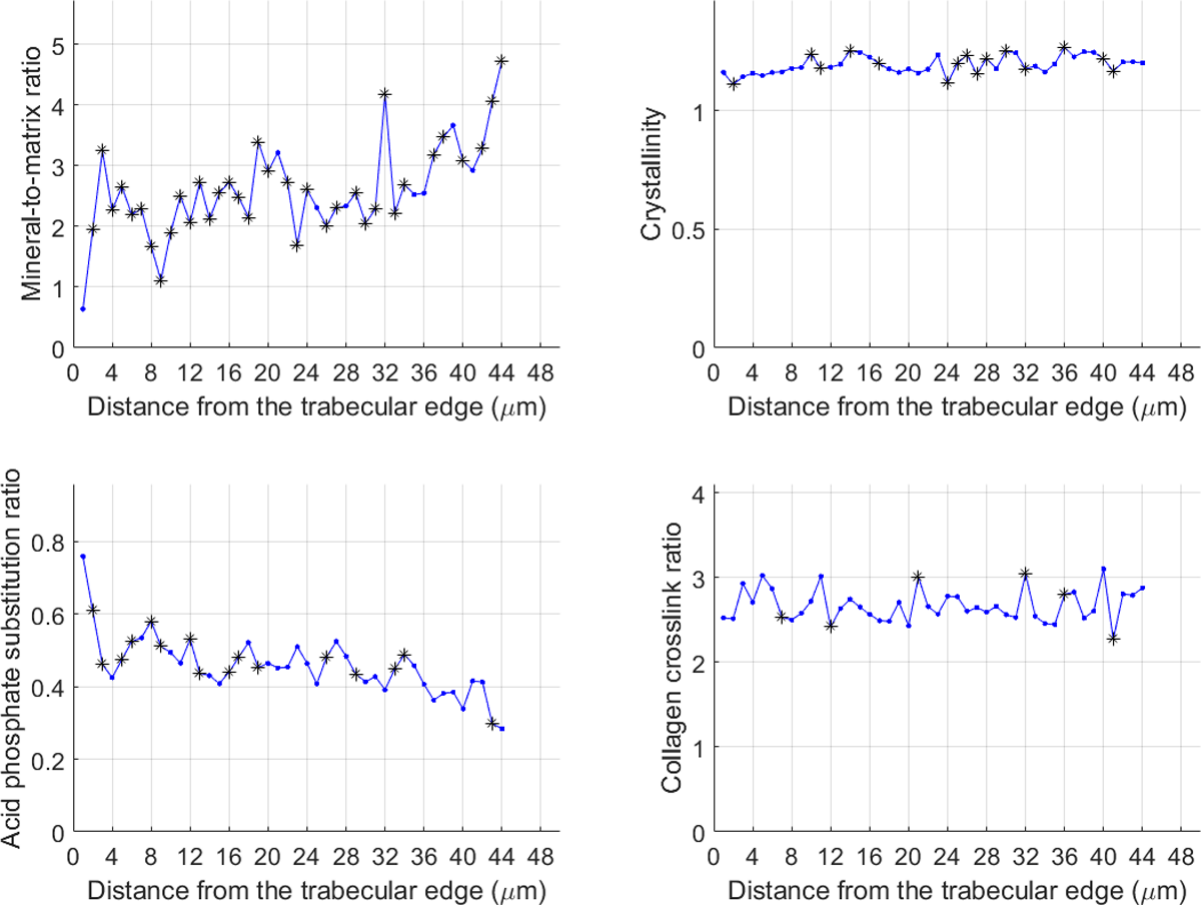
Example of a single line scan recorded by AFM-IR. Evolution of the corresponding infrared parameters as a function of the distance from the trabecular edge resulting from repeated measurements acquired as a line orthogonal to the trabecula edge. * p<0.05 compared to the previous data point. The p-values were calculated using a one-sided Wilcoxon Mann Whitney test.

**Fig 8:**
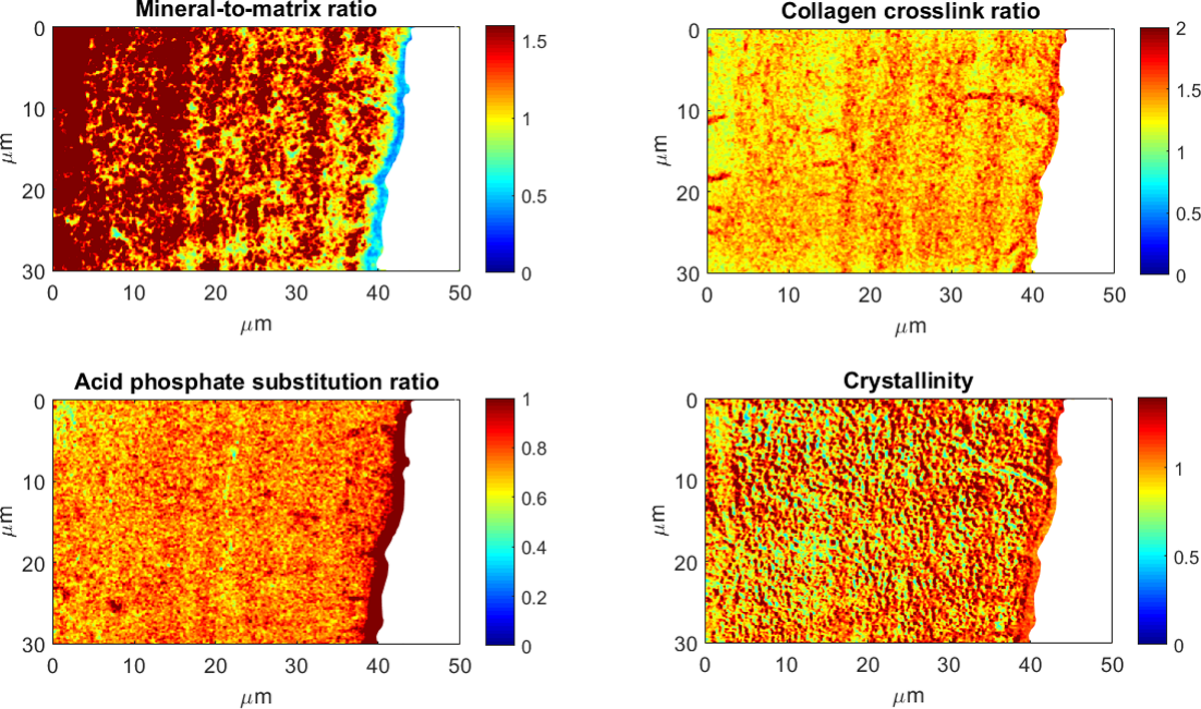
AFM-IR images of the four spectroscopic parameters acquired on the same area of a cancellous bone sample. Maps were derived from images acquired at six wavenumbers: 1128 cm^-1^, 1096 cm^-1^, 1030 cm^-1^, 1020 cm^-1^ (mineral band), 1690 cm^-1^, and 1660 cm^-1^ (amide). Those maps, particularly the mineral-to-matrix ratio and the collagen crosslink ratio, show layers of high and low intensities. Color scales are the AFM-IR intensity ratios.

Reproducible and statistically significant alternating patterns were observed for the mineral-to-matrix ratio and the acid phosphate substitution ratio in the line acquisitions going from the newly formed bone on the surface (younger bone) to the center of the trabecula (more mature bone) (Fig 7). The periodicity was not constant and ranged from 2 to 8 microns. Images acquired at wavenumbers specific to mineral and collagen also showed alternating layers (Fig 8), suggesting compositional and/or structural alternation. Interestingly, topography images (AFM) also showed a structural pattern of layers of different orientations (Fig 5).

**Fig 9:**
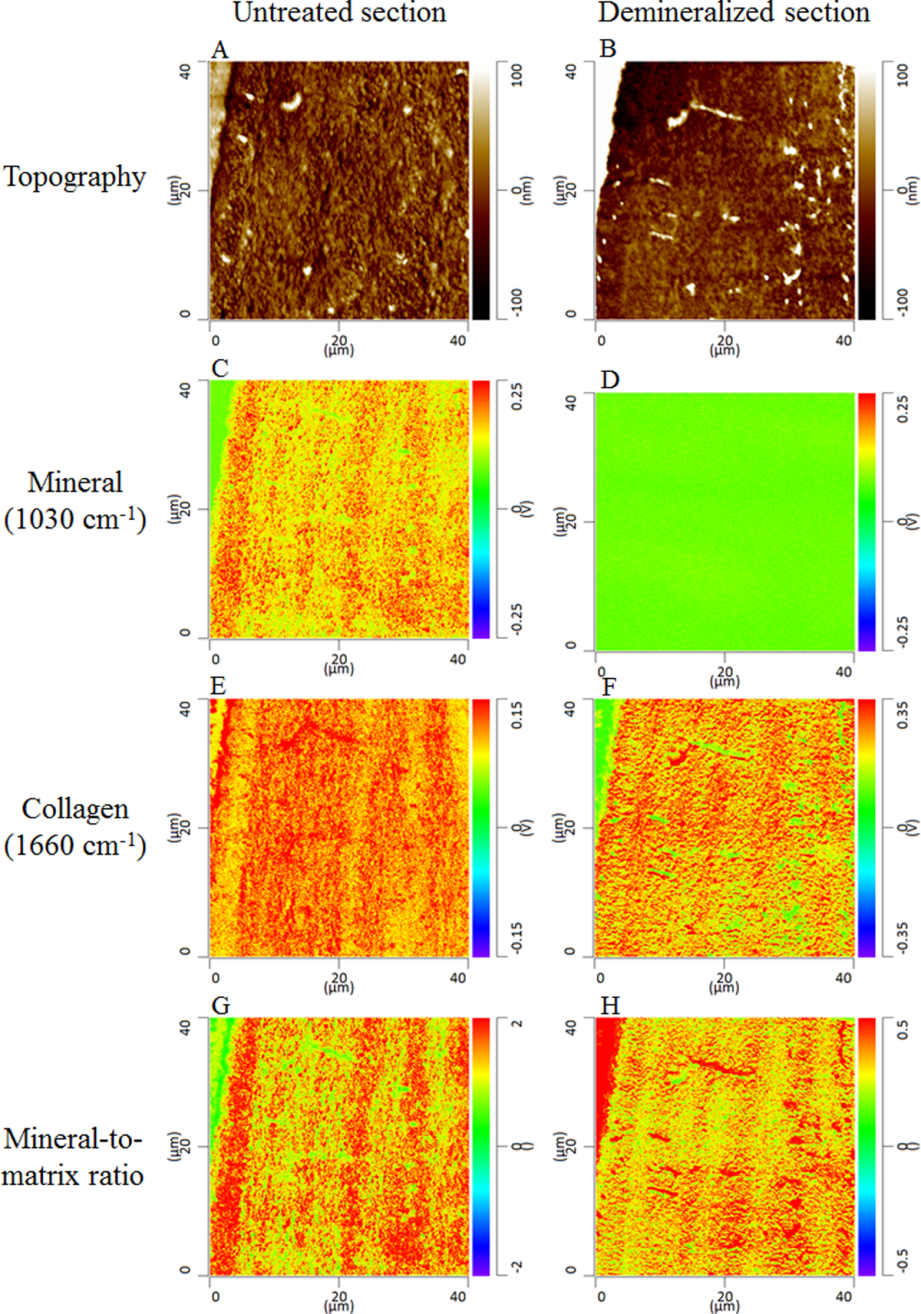
Height and infrared intensities acquired on a cancellous bone thin section (300 nm) before and after demineralization of the section using EDTA. The topographic images (A and B) were acquired with the AFM in contact mode. The mineral (C and D) and collagen (E and F) images were acquired by AFM-IR at wavenumbers 1030 cm^-1^ and 1660 cm^-1^, respectively. The ratio of these two images is the mineral-to-matrix ratio (G and H). Before EDTA treatment the images had an alternating (layered or striated) pattern when both mineral and collagen were present in the section. The two components are entangled, and individual contributions cannot be distinguished. To uncouple the contributions, the collagen component was isolated by dissolving the mineral crystals. After EDTA treatment, the mineral image (D) shows a null intensity, indicating that the demineralization was accomplished. In the collagen image after demineralization (F) an alternating pattern still exists in the absence of mineral, suggesting that the collagen (either structure or density) is a significant contributor to the periodic pattern although the collagen structure may be affected by the mineral dissolution. The collagen pattern is also reflected in the mineral-to-matrix image.

To further understand the contribution of tissue composition to the alternating pattern, we examined the same section with and without mineral. The alternating pattern remained in the mineralized sample even in the absence of mineral (Fig 9). Therefore, the collagen fibers and their organization are major contributors to the alternating pattern in the trabecula.

## Discussion

Bone composition is an important contributor to bone mechanical integrity. FTIR is commonly used to assess bone tissue composition at a resolution of several microns. However, nanoscale analysis techniques are needed because bone ultrastructure exhibits features well below the resolution of FTIR, especially in newly formed bone tissue. In the current study, we validated the temporal and spatial reproducibility of a novel infrared technique, called AFM-IR, on PMMA and compared the spectra to those obtained by traditional FTIR. We also studied cancellous bone composition at the nanoscale with this new technique and highlighted the presence of nanoscale structure.

When comparisons were made between AFM-IR and FTIR spectra, major peak positions were similar for the homogeneous PMMA resin and for bone samples (Fig 3A and 3B). Good agreement was shown previously between AFM-IR and FTIR spectra on polymers [33,44], but the ratios appeared to be different for heterogeneous materials [42], most likely due to scale effects. FTIR measures absorption from the bulk, whereas AFM-IR permits local measurement at higher resolution. In the current study, peak shapes and intensities also varied between the two techniques, suggesting that the parameters calculated from the spectra likely will differ. The shapes of the mineral peaks at the trabecular edge were similar; however, surface pixels are often excluded from the analysis of FTIR images. The spectral variations are suggestive of differences in structure of the mineral crystals and the collagen matrix [41]. In particular, interesting features were evident in the spectra at 1600 cm^-1^ (amide) and 1130 cm^-1^ (mineral). Additional experiments will be needed to fully understand the cause of the shape changes in the amide and mineral bands. By definition, AFM-IR will provide more detailed images on local regions than FTIR. The improved resolution of AFM-IR was particularly evident at the surface of trabeculae. In nanoscale images, a layer without mineral was present juxtaposing the trabecular edge, suggesting the presence of newly formed collagen matrix that had not yet mineralized.

AFM-IR was used to investigate mineralization as a function of bone maturation, defined as distance from the trabecular surface. In the line acquisitions, all samples exhibited reproducible alternating patterns with periodicities of 2 to 8 μm for mineral-related-IR parameters: mineral-to-matrix ratio and acid phosphate substitution ratio. A similar pattern was evident for the mineral-to-matrix ratio in imaging mode. A similar periodicity (6 μm) was previously reported for the elastic properties of osteons, corresponding to the thickness of lamellae [45]. In human cancellous bone, lamellar patterning has been associated with mineralization variability in multiple studies using different techniques that measure the mineral properties [46,47]. This variation of mineralization was hypothesized to modulate the tissue mechanical properties and particularly increase the tissue toughness. However, the focus was on mineral density and the matrix structure was seldom reported.

During in vivo bone development, matrix formation precedes mineral deposition. Collagen content and structure guide the subsequent mineral content and structure. When both matrix and mineral are examined, the orientation of the collagen fibrils explains the modulation in microelastic properties in osteons better than the mineral density [48]. In the present study, this alternating pattern was present in matrix parameters (Fig 8), which was confirmed by analysis of a demineralized section (Fig 9). Therefore, the alternating pattern of the mineral-to-matrix ratio could reflect the alternating pattern of collagen content and / or structure. Numerous studies have observed alternating patterns due to fiber orientation using polarized light microscopy [49], Raman spectroscopy [50,51] associated with acoustic microscopy [52], and synchrotron X-ray phase nanotomography (SR-PNT) [53]. The patterning has also been ascribed to a difference in collagen density [54], whereas Reznikov *et al*. showed that both orientation and density of both components could vary in lamellar bone [55]. Further experiments will be needed to assess the sensitivity of the technique to orientation and to confirm the relative contributions of fiber and mineral orientation and content to the alternating pattern.

Periodic patterns were characterized originally in enamel, which displays both short- and long-term periodic patterns. Daily appositional growth produces cross striations [56]. In addition to this short-range circadian rhythm, enamel also has a long periodic rhythm evident in layers called striae of Retzius, with a repeat interval made of multiples of the shorter-term pattern [57–59]. Rather than a mechanical function, conservation of the short-term pattern and the repeat interval are hypothesized to reflect a central biological timing mechanism [59]. Bone also forms incrementally and displays circadian growth rhythms, in the form of lamellae, on the same order as those observed in enamel [60].

This study is the first to systematically validate AFM-IR and apply the technique to characterize the nanostructure of mineralized tissue. As with many novel techniques, these initial results raise many additional questions. Our initial data are based on parameters that are well-validated for FTIR spectroscopy. Future work should confirm the specific mineral and matrix parameters appropriate to this new technique. We should also consider application to additional mineralized tissues, including enamel, where striated patterns have been well characterized by other techniques.

In conclusion, this study used a novel nanoscale technique, AFM-IR, to investigate cancellous bone tissue composition at 50–100 nm resolution. After verifying the reproducibility on a homogeneous material (PMMA), we compared the novel technique with traditional FTIRI. The repeated measurements and images showed variations of AFM-IR parameters as a function of tissue maturity, as expected from the literature; in particular, the mineral-to-matrix ratio and the acid phosphate substitution ratio increased and decreased, respectively, as a function of bone maturity. AFM-IR identified an alternating pattern that we propose corresponds to the lamellar structure. In addition to further technique development, future work will examine the relative roles of the matrix structure and mineral content in the underlying pattern seen in cancellous bone.
